# TGF-*β*1 genotype and phenotype in breast cancer and their associations with IGFs and patient survival

**DOI:** 10.1038/sj.bjc.6604689

**Published:** 2008-09-30

**Authors:** L Mu, D Katsaros, L Lu, M Preti, A Durando, R Arisio, H Yu

**Affiliations:** 1 Department of Epidemiology and Public Health, Yale Cancer Center, Yale University School of Medicine, New Haven, CT 06520-8034, USA; 2 Department of Obstetrics and Gynecology, Gynecologic Oncology and Breast Cancer Unit, University of Turin, Turin, Italy; 3 Department of Pathology, S’Anna Hospital, Turin, Italy

**Keywords:** TGF-*β*1, polymorphism, IGFs, breast cancer, survival

## Abstract

Transforming growth factor-*β* (TGF-*β*)-mediated signals play complicated roles in the development and progression of breast tumour. The purposes of this study were to analyse the genotype of *TGF-β1* at T29C and TGF-*β*1 phenotype in breast tumours, and to evaluate their associations with IGFs and clinical characteristics of breast cancer. Fresh tumour samples were collected from 348 breast cancer patients. TGF-*β*1 genotype and phenotype were analysed with TaqMan® and ELISA, respectively. Members of the IGF family in tumour tissue were measured with ELISA. Cox proportional hazards regression analysis was performed to assess the association of TGF-*β*1 and disease outcomes. Patients with the T/T (29%) genotype at T29C had the highest TGF-*β*1, 707.9 pg mg^−1^, followed by the T/C (49%), 657.8 pg mg^−1^, and C/C (22%) genotypes, 640.8 pg mg^−1^, (*P*=0.210, T/T *vs* C/C and C/T). TGF-*β*1 concentrations were positively correlated with levels of oestrogen receptor, IGF-I, IGF-II and IGFBP-3. Survival analysis showed TGF-*β*1 associated with disease progression, but the association differed by disease stage. For early-stage disease, patients with the T/T genotype or high TGF-*β*1 had shorter overall survival compared to those without T/T or with low TGF-*β*1; the hazard ratios (HR) were 3.54 (95% CI: 1.21–10.40) for genotype and 2.54 (95% CI: 1.10–5.89) for phenotype after adjusting for age, grade, histotype and receptor status. For late-stage disease, however, the association was different. The T/T genotype was associated with lower risk of disease recurrence (HR=0.13, 95% CI: 0.02–1.00), whereas no association was found between TGF-*β*1 phenotype and survival outcomes. The study suggests a complex role of TGF-*β*1 in breast cancer progression, which supports the finding of *in vitro* studies that TGF-*β*1 has conflicting effects on tumour growth and metastasis.

Transforming growth factor-*β* (TGF-*β*) is a group of multifunctional proteins, which include three TGF-*β* isoforms (TGF-*β*1, -*β*2, -*β*3) and several other polypeptides. These growth factors regulate important cellular activities, including cell proliferation, differentiation, apoptosis and motility, and the formation of extracellular matrix. The actions of TGF-*β* are mediated through a cell membrane receptor TGF-*β* receptor II (T*β*RII), which, upon binding to its ligand, recruits another TGF-*β* receptor (T*β*RI) forming a receptor heterodimer. The formation activates the receptor's serine/threonine kinase, which phosphorylates Smad2 or Smad3. The phosphorylated Smad2/3 forms a complex with Smad4 which moves to the nucleus and binds to DNA targets initiating complicated TGF-*β* effects (Dumont and Arteaga, 2000; Wakefield *et al*, 2001; Buck and Knabbe, 2006; Chang *et al*, 2007; Cox *et al*, 2007).

Evidence suggests that TGF-*β*-mediated signals are important in the development and growth of breast tissues (Wakefield *et al*, 2001). TGF-*β* is involved in the formation of mammary gland architecture, regulation of stem cell kinetics, maintenance of mammary epithelium in a functionally undifferentiated state, and initiation of apoptosis in gland involution. Both *in vitro* and *in vivo* experiments have shown that TGF-*β* inhibits the proliferation of mammary epithelial cells. In TGF-*β*1 and TGF-*α* double-transgenic mice, tumour occurrence was significantly reduced compared to the animals with TGF-*α* transgene alone. In addition, mammary gland tumorigenesis induced by 7, 12-dimethybenz[*α*] anthracene was prevented by TGF-*β* expression. TGF-*β* induces premature senescence of mammary stem cells and thus may suppress tumour development (Pierce *et al*, 1995; Siegel and Massague, 2003). Evidence, however, also suggests that TGF-*β* may stimulate tumorigenesis in epithelial cells through inducing epithelial-to-mesenchymal transition, disrupting cell adhesion, and enhancing cell invasiveness (Miettinen *et al*, 1994; Oft *et al*, 1996; Knudsen *et al*, 1998; Blobe *et al*, 2000). TGF-*β*1 upregulates integrin-linked kinase, and increases cell motility. Recent studies have shown that alterations in TGF-*β* signalling have biphasic effects on tumour progression and metastasis (Tang *et al*, 2003; Serra and Crowley, 2005; Chang *et al*, 2007).

TGF-*β*1 is the most abundant TGF-*β* in many tissues. The *TGF-β1* gene is located on chromosome 19q13, and is expressed as a 25-kDa protein in endothelial and haematopoietic cells, as well as in connective tissues (Chang *et al*, 2007). Several genetic polymorphisms exist in the *TGF-β1* gene, one of which is a T → C transition at nucleotide 29 of the coding region, resulting in a leucine to proline substitution at codon 10 (Blobe *et al*, 2000; Breast Cancer Association Consortium, 2006; Cox *et al*, 2007). This single nucleotide polymorphism (SNP) T29C, which is in strong linkage disequilibrium with another common SNP C-509T, has been studied extensively for its association with breast cancer risk and survival (Yokota *et al*, 2000; Goode *et al*, 2002; Hishida *et al*, 2003; Krippl *et al*, 2003; Ziv *et al*, 2003; Jin *et al*, 2004; Le Marchand *et al*, 2004; Shu *et al*, 2004; Kaklamani *et al*, 2005; Lee *et al*, 2005; Shin *et al*, 2005; Skerrett *et al*, 2005; Breast Cancer Association Consortium, 2006; Feigelson *et al*, 2006; Gonzalez-Zuloeta Ladd *et al*, 2007). Two studies found increased risk of breast cancer among Koreans and Dutch who carry the C allele (Lee *et al*, 2005; Gonzalez-Zuloeta Ladd *et al*, 2007), whereas two other studies showed decreased risk in relation to C genotype among Americans who were older than 65 years or Japanese who were premenopausal (Hishida *et al*, 2003; Ziv *et al*, 2003). Another study conducted in China observed the association varied by disease stage (Shin *et al*, 2005). Reduced disease-free survival in patients who carried the C allele was observed in some (Shu *et al*, 2004; Skerrett *et al*, 2005), but not other studies (Goode *et al*, 2002; Gonzalez-Zuloeta Ladd *et al*, 2007). The C allele at T29C has been associated with high levels of circulating TGF-*β*1 in a Japanese study (Yokota *et al*, 2000), but no studies have evaluated this relationship in breast tissue.

Associations of TGF-*β*1 concentrations in tumour tissue and breast cancer survival have been examined in a number of studies, and the results were inconsistent. Several studies found high TGF-*β*1 being associated with poor survival (Gorsch *et al*, 1992; Walker and Dearing, 1992; Dalal *et al*, 1993; Walker *et al*, 1994; Desruisseau *et al*, 2006), whereas others showed an opposite association (Murray *et al*, 1993; Kesari *et al*, 1999). Most of the studies used semi-quantitative immunohistochemical staining to assess TGF-*β*1 levels in breast tumours. There was only one study using ELISA to measure TGF-*β*1 in tissue samples, and the study found high TGF-*β*1 in association with poor survival in node-negative, but not in node-positive patients (Desruisseau *et al*, 2006). Studies also suggest possible synergies between TGF-*β* and insulin-like growth factors (IGFs) (Tsukazaki *et al*, 1994; Wong *et al*, 2001). To further elucidate the role of TGF-*β*1 in breast cancer, we analysed TGF-*β*1 genotype at T29C and protein levels in tumour samples, and examined the associations of these markers with IGFs, clinical characteristics of breast cancer and patient survival.

## Materials and methods

### Study subjects

Patients who underwent surgery for beast cancer in the Gynecologic Oncology Unit at University of Turin in Italy between January 1998 and July 1999 were recruited for the study, which was approved by the university’s ethical review committee. During the study, fresh tumour samples were collected from 348 patients; the specimens were snap-frozen in liquid nitrogen immediately after resection and then stored at −80°C until analysis. Clinical information collected for the study includes age at surgery, disease stage, tumour grade, tumour size, number of lymph nodes tested positive for cancer, histological type and hormone receptor status. Of the 348 patients, 302 were followed from surgery through February 2007; during the follow-up, information on relapse and death was collected.

### Tumour tissue analysis

Frozen tumour samples were pulverised manually in liquid nitrogen. Genomic DNA was extracted from approximately 100 mg of tissue powder following a standard phenol–chloroform protocol. SNP genotype at T29C of *TGF-β1* was analysed with a TaqMan assay (PN: C_22272997, Applied Biosystems, Foster City, CA, USA). The PCR procedure consisted of initial denaturing at 95°C for 10 min, and 50 cycles of denaturing at 92°C for 15 s and annealing/extension at 68°C for 1 min. The PCR was carried out in the ABI 7500 system with about 10 ng of genomic DNA.

Tissue proteins were extracted from approximately 100 mg of tissue powder using a commercial kit (Clonetech, Palo Alto, CA, USA). The extraction was done through mixing the tissue power with 1 ml BD TALON × Tractor buffer, and collecting supernatant after centrifuging the mixture at 14 000 r.p.m. for 30 min at 4°C. Concentrations of total protein in tissue extracts were measured using the bicinchoninic acid method (Pierce Inc., Rockford, IL, USA). A commercial ELISA kit for human TGF-*β*1 (R&D Systems Inc., Minneapolis, MN, USA) was used to determine TGF-*β*1 concentration in tumour samples (DB100B). The assay was performed following the manufacturer’s instruction. The amount of TGF-*β*1 in tumour samples was measured after the latent TGF-*β*1 was activated by acid-treatment (activation by 1N HCI and neutralisation by 1.2N NaOH). All the specimens were tested in duplicate after the 2-fold dilution. If the concentration of TGF-*β*1 exceeded the highest standard after dilution, the sample was further diluted and re-measured. The measurement was repeated if the CV (coefficient of variation) on the duplicate was greater than 10%. The final concentration of TGF-*β*1 in each sample was adjusted for total protein. Tissue levels of IGF-I, IGF-II and IGFBP-3 were analysed in the same samples for a previous study of IGFs (Mu *et al*, 2008). Four commercial ELISA kits (Diagnostic Systems Laboratories, Webster, TX, USA) were used to measure the concentrations of IGF-I, free-IGF-I, IGF-II and IGFBP-3 in tumour samples, and the measurement was carried out in the same manner as that for TGF-*β*1 described above. Tumour hormone receptor status, including oestrogen receptor (ER) and progesterone receptor (PR), was measured routinely in the clinic to assist post-operative treatment.

### Statistic analysis

TGF-*β*1 genotype and phenotype were compared among patients with different clinical and pathological features using the Wilcoxon rank sum test or *χ*^2^ test where appropriate. Correlations of TGF-*β*1 protein with numerical or ordinal variables were analysed using the Spearman correlation coefficient. Cox proportional hazard regression models were employed for survival analysis. In the analysis, tissue levels of TGF-*β*1 were grouped into low, medium and high categories based on their tertile distribution. Low category was used as reference in data analysis. Disease-free survival was the time interval from the date of surgery to date of recurrence or last follow-up. Overall survival was the time between the date of surgery and date of breast cancer death or last follow-up. Hazard ratios and their 95% confidence intervals were calculated at both univariate and multivariate levels. Age at surgery, TNM stage, tumour grade, histological type, oestrogen receptor and progesterone receptor were included in the multivariate analysis.

## Results

### TGF-*β*1 genotype and phenotype in breast cancer

**Figure 1:**
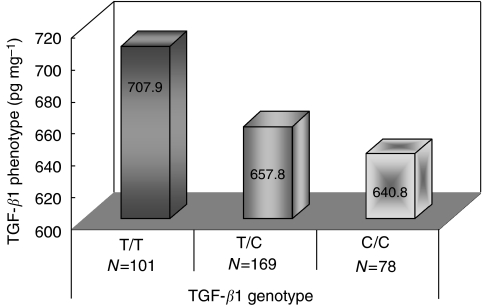
TGF-*β*1 genotype and phenotype in breast tumour tissue.

For the TGF-*β*1 genotype at T29C, 101 patients (29%) carried the T/T genotype, 78 (22%) had the C/C genotype, and 169 (49%) were heterozygous T/C. TGF-*β*1 protein was detectable in all the tumour samples, and the median concentration was 662.2 pg mg^−1^, ranging from 56.6 to 3640.4 pg mg^−1^. Figure 1 shows the level of TGF-*β*1 in tumour tissue by different genotypes of *TGF-β1* at T29C. The highest level of TGF-*β*1 was seen in the T/T genotype (median: 707.9 pg mg^−1^). The average TGF-*β*1 levels were 657.8 and 640.8 pg mg^−1^ in the T/C and C/C carriers, respectively. The differences in tissue levels of TGF-*β*1 by the genotype, however, were not statistically significant (*P*=0.210).

### Associations of TGF-*β*1 with disease characteristics and IGF expression

Comparisons of TGF-*β*1 genotype and phenotype over different disease characteristics are shown in Table 1. No significant association was observed between TGF-*β*1 genotype and clinical characteristics of breast cancer. However, TGF-*β*1 protein levels were significantly different by TNM stage and ER status. Higher TGF-*β*1 was found in early-stage disease (Stage I/II) or ER-positive tumours. Levels of TGF-*β*1 were significantly correlated with disease stage (*r*=−0.14) and ER status (*r*=0.18) (Table 2). Peptide levels of IGF markers measured in our previous study (Mu *et al*, 2008) were also evaluated for their relationship with TGF-*β*1. The analysis showed that the protein was positively correlated with IGF-I (*r*=0.13), IGF-II (*r*=0.33) and IGFBP-3 (*r*=0.23) (Table 2).

### TGF-*β*1 genotype and phenotype in relation to patient survival

Table 3 shows the associations of TGF-*β*1 genotype and phenotype with disease-free and overall survival (DFS and OS). When analysing all patients together, we found no significant associations between DFS and TGF-*β*1, either genotype or phenotype. However, OS was associated with TGF-*β*1 genotype, but not phenotype. Patients with the T/T genotype had more than 2-fold increases in risk of breast cancer death (*P*=0.013). This association was more evident among patients with early-stage disease. Both TGF-*β*1 genotype and phenotype were associated with OS. Patients with stage I or II breast cancer had 4-fold increases in risk of death if they had the T/T genotype compared with C/C (*P*=0.002) or 2-fold increases in risk of death if they had high compared with low TGF-*β*1 (*P*=0.030). The trends for increasing risk of death from the C/C to C/T to T/T genotype or from low to median to high TGF-*β*1 were also significant, and remained significant after adjusting for age, grade, histology and ER/PR status (Table 3). Similar associations, however, were not observed for patients with late-stage disease. Among patients with stage III or IV disease, the risk for relapse or death was lower in the T/T genotype compared with C/C genotype or lower in high compared with low TGF-*β*1, although most of the associations were not statistically significant except for one.

## Discussion

The distribution of the T29C genotype in our study population was quite similar to those reported in Polish (Jin *et al*, 2004), African American and Latino (Le Marchand *et al*, 2004), but the C/C genotype in our study was slightly higher than that in Finnish, German and Dutch (Jin *et al*, 2004), and relatively lower than that in Japanese, Hawaii and Chinese (Le Marchand *et al*, 2004). We found no significant association of this polymorphism with breast cancer progression when analysing all the patients together. However, the results were different when the analysis was stratified by disease stage. The T/T genotype was associated with increased risk of relapse in patients with early-stage disease, but not in patients with late-stage disease. Consistent with the finding of disease-free survival, overall survival was also associated with the TGF-*β*1 genotype. Patients with the T/T genotype had significantly higher risk of death than those without the genotype. The elevated risk was more evident in patients with early-stage disease, and remained significant after adjusting for clinical and pathological factors. More importantly, similar associations were also observed for the TGF-*β*1 phenotype. High levels of TGF-*β*1 protein, which were in the same direction as the T/T genotype, were associated with increased risk of disease progression and death, and the association was especially substantial for overall survival and was independent of other factors.

These associations, however, were not observed in patients with late-stage disease. In fact, the associations of TGF-*β*1 with disease progression and death in late-stage patients were in an opposite direction; patients with the genotype and phenotype of high TGF-*β*1 seemed to have reduced risk of disease progression or death, although most of the associations were not statistically significant. The apparently opposite relationship between TGF-*β*1 and disease progression suggests that TGF-*β*1 may have different effects on breast cancer progression depending on the stage of the disease. The effect of TGF-*β*1 on breast cancer survival has been evaluated by several previous studies, and the findings are quite inconsistent. Two studies observed shorter survival among the C allele carriers (Shu *et al*, 2004; Skerrett *et al*, 2005), whereas one showed longer survival (Gonzalez-Zuloeta Ladd *et al*, 2007). Another study found no association (Goode *et al*, 2002). Of these studies, none has analysed the association by disease stage. Interestingly, our results seem to be in agreement with those of the studies, which evaluated the effect of TGF-*β*1 genotype on breast cancer risk. Two studies found that women with TGF-*β*1 C allele had lower risk for early-stage breast cancer, but higher risk for late-stage disease (Shin *et al*, 2005; Feigelson *et al*, 2006).

Both *in vitro* and *in vivo* experiments have shown that TGF-*β*1 is expressed in breast cancer. Our study also demonstrated that TGF-*β*1 was detectable in breast tumours by ELISA, and the median concentration was 662.2 pg mg^−1^. Furthermore, we found that TGF-*β*1 levels were slightly different by the TGF-*β*1 genotype at T29C with low concentrations in the C allele carriers. This finding was in agreement with a previous study in which the C/C genotype was called a low production genotype and the T/T genotype was a high production one (Skerrett *et al*, 2005). We also found in the study that TGF-*β*1 levels in tumour tissues were positively correlated with the concentrations of IGF-I, IGF-II, and IGFBP-3. These IGF markers were analysed in our previous study where we found no indications of associations between tissue levels of IGF peptides and disease-free or overall survival among the same patients (Mu *et al*, 2008). The correlations between TGF-*β*1 and IGFs may suggest a potential synergy between these growth factors or a shared or related mechanism involving the regulation of these molecules. Synergistic interaction between TGF-*β*1 and IGFs was suggested by an *in vitro* experiment (Tsukazaki *et al*, 1994). TGF-*β*1 and IGF-I were found to work in concert in regulating the activity of an oestrogen-metabolising enzyme called estradiol-17-*β* hydroxysteroid dehydrogenase in MCF-7 cells (Wong *et al*, 2001). Jointly elevated TGF-*β*1 and IGF-I were also seen in sera and local tissues under other pathological conditions (Li *et al*, 2002; Stracke *et al*, 2002). Thus, our finding of positive correlations between TGF-*β*1 and IGFs in breast cancer does not seem to be unexpected, and it may have some biologic implications in the disease.

Our survival analysis suggests that the effect of TGF-*β*1 on breast cancer progression may be quite different depending on the stage of the disease. High TGF-*β*1 was associated with increased risk of disease progression and death among patients with early-stage disease, but with decreased risk among patients with late-stage disease. Similar results were also observed in a previous study, which was the only study that quantitatively measured TGF-*β*1 levels in breast tumours using ELISA. That study found high TGF-*β*1 associated with poor DFS in node-negative, but not in node-positive patients. It also observed TGF-*β*1 levels in association with disease characteristics depending on nodal status. ER-negative tumours had lower TGF-*β*1 than ER-positive ones if patients were node-negative. However, for node-positive patients, ER-positive tumours had lower TGF-*β*1 than ER-negative ones (Desruisseau *et al*, 2006). These findings seem to support the possibility that TGF-*β*1 may have different functions in breast cancer progression depending on the stage of the disease.

It is known that the actions of TGF-*β*1 differ substantially by its location. Although cellular TGF-*β*1 inhibits tumour growth, TGF-*β*1 in extracellular matrix stimulates tumour cell motility and migration, facilitating cancer invasion and metastasis (Arteaga *et al*, 1990; Reiss, 1999; Barcellos-Hoff, 2005). In mammary gland, TGF-*β*1 is a potent inhibitor of cell proliferation, and in certain circumstances it can also induce apoptosis (Silberstein and Daniel, 1987; Nguyen *et al*, 2000). Studies have shown that when tumour develops, the expression of T*β*RII by breast cancer cells is diminished, which results in reduced response of tumour cells to TGF-*β*1 signalling and increased secretion of the ligand (Akhurst and Balmain, 1999; Reiss, 1999). Under this situation, the inhibitory effect of TGF-*β*1 on tumour cells is diminished or abrogated. Studies of TGF-*β*1 signal transduction provided further evidence that low expression of T*β*RII by breast cancer cells was correlated with enhanced *in vivo* malignant behaviours both in tumour specimens and a cancer cell line (Gobbi *et al*, 1999). Tobin *et al*, (2002) observed a marked enhancement of tumour growth *in vivo* by tumour-derived TGF-*β* when an autocrine loop was left intact. Studies also found that increased production of TGF-*β*1 by tumour cells was associated with potential invasiveness and metastasis of cancer (Gorsch *et al*, 1992; Dalal *et al*, 1993; Ueki *et al*, 1993; Tobin *et al*, 2002).

It was noted that tumour cells with increased production of TGF-*β*1, which could inhibit tumour cell growth *in vitro*, stimulated vigorous tumour growth *in vivo* when the cells were inoculated into an animal model (Steiner and Barrack, 1992; Chang *et al*, 1993). The conflicting results suggest the effect of TGF-*β*1 may be regulated by multiple mechanisms, and the tumour–host interaction may play an important role in tumour growth. TGF-*β* signalling appeared to have a dual role in mammary tumour progression in transgenic mice. In Neu transgenic mice, constitutively active T*β*R1 prolonged the latency of mammary tumour formation, but enhanced the frequency of lung metastasis. In contrast, dominant-negative T*β*RII reduced tumour metastasis in Neu mice, but shortened the median latency of tumours induced by polyomavirus middle-T. It was also reported that T*β*RII inactivation increased the invasiveness of premalignant or low-grade breast tumours, but reduced the metastasis of high-grade tumours (Tang *et al*, 2003).

To conclude, the study results suggest that TGF-*β*1 may play a biphasic role in breast cancer progression depending on the stage of the disease. High TGF-*β*1 in early-stage disease may indicate the lack of TGF-*β*1 inhibition because of loss of receptors, which results in unfavourable prognosis. High TGF-*β*1 may also facilitate tumour metastasis and invasion leading to increased risk of disease progression and death.

## Figures and Tables

**Table 1:** Associations of TGF-*β*1 genotype and phenotype with clinicopathological characteristics of breast cancer

		**TGF-*β*1 genotype**			**TGF-*β*1 phenotype**
**Variables**	***N***	**T/T**	**T/C**	**C/C**	**Median (pg mg^−1^)**
Total	347	101 (29.0%)	169 (48.6%)	78 (22.4%)	662.2 (485.7, 942.2)^a^
					
*Age (year)*					
<50	111	21	58	32	653.9
50∼65	139	30	69	40	640.8
65∼	98	27	42	29	669.3
*P-value*			0.589		0.562
					
*Histological type*					
Ductal	230	46	113	71	712.8
Lobular	75	17	39	19	574.7
Others	42	15	17	10	649.8
*P-value*			0.224		0.135
					
*Tumour size (cm)*					
⩽2	206	41	111	54	667.6
>2	140	37	57	46	640.1
*P-value*			0.054		0.798
					
*Lymph node*					
Negative	183	42	87	54	659.5
Positive	159	35	78	46	673.3
*P-value*			0.959		0.468
					
*Tumour grade*					
G1/2	199	48	98	53	680.3
G3	144	30	67	47	611.4
*P-value*			0.459		0.153
					
*TNM stage*					
Stage I-II	310	67	154	89	674.5
Stage III-IV	31	10	10	11	497.1
*P-value*			0.163		0.010
					
*Oestrogen receptor (%)*					
<10	126	20	62	44	558.2
⩾10	222	58	107	57	717.6
*P-value*			0.046		0.001
					
*Progesterone receptor (%)*					
<10	169	41	72	56	658.8
⩾10	179	37	97	45	665.7
*P-value*			0.090		0.574

a Range: 25–75%.

**Table 2:** The Spearman correlations of TGF-*β*1 protein, IGF peptide, and breast cancer characteristics

**Disease variable**	**Age**	**Tumour size**	**Lymph node**	**Grade**	**TNM stage**	**ER**	**PR**	**IGF-I peptide**	**Free-IGF-I peptide**	**IGF-II peptide**	**IGFBP-3 protein**
TGF-*β*1 Protein	0.04	−0.03	0.01	−0.06	−0.14^*^	0.18^**^	0.05	0.13^*^	−0.04	0.33^***^	0.23^***^

^*^P<0.05, ^**^P<0.01, ^***^P<0.001.

**Table 3:** TGF-*β*1 genotype, phenotype and breast cancer survival

	**Disease-free survival**		**Overall survival**	
**TGF-*β*1**	**HR (95%CI)**	**AHR (95%CI)** a	**HR (95%CI)**	**AHR (95%CI)** a
*TGF-β1 genotype*				
*Total*				
C/C	1	1	1	1
T/C	0.75 (0.41–1.36)	0.78 (0.43–1.43)	1.01 (0.46–2.21)	1.20 (0.54–2.67)
T/T	1.54 (0.86–2.76)	1.14 (0.62–2.09)	2.21 (1.04–4.72)	1.87 (0.86–4.08)
*P*_trend_	0.063	0.511	0.013	0.079
				
*Stage I/II*				
C/C	1	1	1	1
T/C	1.05 (0.50–2.18)	1.03 (0.49–2.16)	1.79 (0.60–5.32)	1.87 (0.62–5.63)
T/T	2.20 (1.06–4.47)	1.79 (0.86–3.73)	4.03 (1.39–11.69)	3.54 (1.21–10.40)
*P*_trend_	0.010	0.059	0.002	0.007
				
*Stage III/IV*				
C/C	1	1	1	1
T/C	0.53 (0.15–1.83)	0.38 (0.08–1.74)	0.70 (0.17–2.94)	1.42 (0.26–7.71)
T/T	0.53 (0.17–1.68)	0.13 (0.02–1.00)	0.77 (0.20–2.88)	0.82 (0.17–3.97)
*P*_trend_	0.278	0.043	0.686	0.837
				
*TGF-β1 phenotype*				
*Total*				
Low	1	1	1	1
Medium	0.87 (0.50–1.50)	0.96 (0.54–1.68)	0.99 (0.51–1.94)	1.06 (0.54–2.10)
High	1.14 (0.68–1.93)	1.23 (0.72–2.12)	1.34 (0.70–2.53)	1.41 (0.73–2.72)
*P*_trend_	0.624	0.449	0.370	0.303
				
*Stage I/II*				
Low	1	1	1	1
Medium	1.06 (0.56–2.02)	1.24 (0.64–2.42)	1.69 (0.72–3.98)	1.80 (0.76–4.29)
High	1.55 (0.85–2.85)	1.78 (0.94–3.37)	2.43 (1.07–5.51)	2.54 (1.10–5.89)
*P*_trend_	0.140	0.069	0.030	0.027
				
*Stage III/IV*				
Low	1	1	1	1
Medium	0.53 (0.17–1.70)	0.29 (0.06–1.45)	0.34 (0.07–1.59)	0.66 (0.09–4.72)
High	0.32 (0.07–1.49)	0.24 (0.04–1.25)	0.21 (0.03–1.67)	0.19 (0.02–1.68)
*P*_trend_	0.106	0.076	0.071	0.120

a Adjusted Hazard Ratio, adjusting for age, grade, TNM stage (only adjusted when all subjects were analysed), histotype, ER, PR.
